# Genome Sequence of *Hafnia alvei* bta3_1, a Bacterium with Antimicrobial Properties Isolated from Honey Bee Gut

**DOI:** 10.1128/genomeA.00439-16

**Published:** 2016-06-09

**Authors:** Baoyu Tian, Nancy A. Moran

**Affiliations:** a College of Life Science, Fujian Normal University, Fuzhou, Fujian, China; b Department of Integrative Biology, University of Texas, Austin, Texas, USA

## Abstract

*Hafnia alvei* bta3_1, a strain with antibacterial properties, was isolated from honey bee gut and cultured under aerobic and anaerobic conditions. To explore the potential genetic bases of its antibacterial and possible pathogenic properties, the complete genome of this organism was sequenced and analyzed.

## GENOME ANNOUNCEMENT

Honey bees (*Apis mellifera*) are important agricultural pollinators (1) that have suffered declines in recent years (2). Microbial pathogens contribute to these declines, emphasizing the need to better understand microbial associates of honey bees. *Hafnia alvei* is one of several *Enterobacteriaceae* species that occur sporadically in bee gut and that may represent opportunistic pathogens. We sequenced and analyzed the genome of *H. alvei* bta3_1, a Gram-negative, facultatively anaerobic, rod-shaped bacterium isolated from honey bee gut. *H. alvei* bta3_1 showed antagonistic activity toward a tested strain of *Bacillus* sp., but no activity was detected toward *E. coli* EPI300, *Paenibacillus larvae*, or fungal strains.

DNA from *H. alvei* bta3_1 (3), was used to make a mate-pair library with average insert size of 2,933 bp (500 8,000 bp) that was end sequenced with Illumina HiSeq2000, resulting in 67,735,948 reads with average length of 76 bp. Ten percent of trimmed, filtered reads were randomly extracted and *de novo* assembled using CLC Genomic Workbench 4.6.1 with an optimal k-mer value of 43, yielding 291 contigs (>200 bp) of average 27.5-fold coverage. SSPACE (4) and Bowtie 2 2.0.2 (5) were used for scaffolding the pre-assembled contigs, and scaffolds were visualized using samtools 0.1.18 (6) and Tablet 1.12.09.03 (7), resulting in 8 super-scaffolds disrupted by rRNA operons. These were organized into linear draft genome sequences using mate-pair information. The gap-filled genomic sequences were manually revised using Tablet to finalize the consensus linear genome. Annotation was carried out using RAST (8), followed with manual curation by searching the automatically annotated coding sequences against GenBank nr and Pfam databases. Phylogenomic analyses were carried out using the core genomic data from 22 *Enterobacteriales* genomes produced in MicroScope (https://www.genoscope.cns.fr). A phylogenomic tree was constructed using the JC model calculated from ProtTest 3.0 (9) and bootstrap replicate branch supports were obtained using PhyML 3.0 in Seaview 4 platform (10).

The final assembly yielded a circular chromosome of 4,763,672 bp and 48.3% G+C. The genome contains 4,363 genes, including 4,270 protein-coding sequences, 72 tRNA genes, and 7 complete rRNA loci. Putative gene clusters were identified for the production of colicin and tolerance to colicin, and siderophores. The presence of genes for production of bacteriocin and siderophore suggests that *H. alvei* can suppress or compete with other microbes inhabiting bee gut. Also present were genes for quorum sensing, biofilm formation, and motility (11–13). Phylogenomic analysis of *Hafnia* and 22 other *Enterobacteriaceae* based on 178 core genes clustered *H. alvei* bta3-1, *H. alvei* FB1, H*. alvei* ATCC 51873, and *Enterobacteriaceae* bacterium 9_2_54FAA together, with *Serratia* as the sister clade. Strain BIDMC_31, which was previously categorized as *H. alvei*, clustered with *Klebsiella*.

*H. alvei* bta3_1 isolated from honey bee gut with antimicrobial activity toward *Bacillus* sp. may represent an opportunistic gut pathogen in honey bees, and potentially interacts with other bacteria in the honey bee gut microbiota.

### Nucleotide sequence accession numbers.

The *H. alvei* bta3_1 genome project has been submitted to GenBank under project accession number PRJNA182589. The assembled and annotated *H. alvei* bta3_1 chromosome has been deposited in GenBank under accession number CP004083.
